# Supplementary material for: Do bed bugs transmit human viruses, or do humans spread bed bugs and their viruses? A worldwide survey of the bed bug RNA virosphere
Source: Virus Res. 2024 Mar 7;343:199349. doi: 10.1016/j.virusres.2024.199349 (PMC10982078; doi:10.1016/j.virusres.2024.199349)
Supplement: Supplementary file 1 [file mmc1.pdf]

SUPPLEMENTARY DATA:

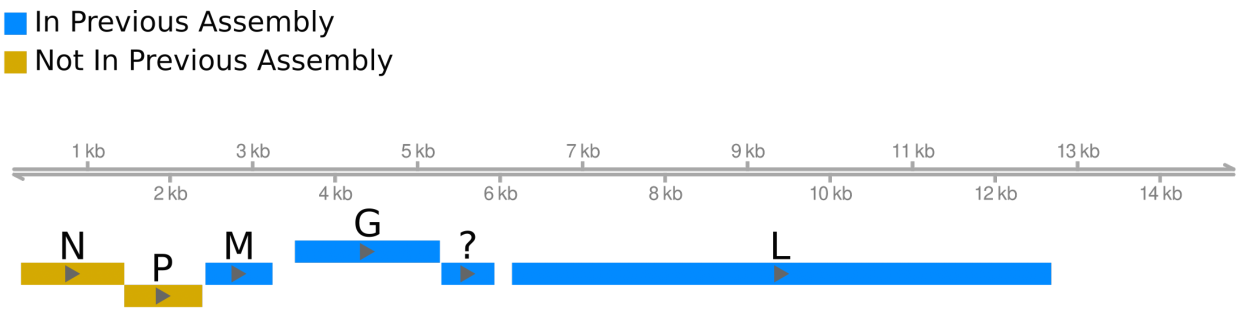

Supplementary Figure 1: Completion of Shuangao bedbug virus 2 (Sbbv2) genome. The previous assembly of Sbbv2 (Refseq: GCF_001755405.1) was missing the nucleoprotein and phosphoprotein typically found in rhabdovirus genomes (shown in blue). Our assembly was around 3 kb longer than the original assembly and included two extra ORFs predicted to be the nucleoprotein and the phosphoprotein (shown in yellow).
